# Abdominal Necrotizing Fasciitis Secondary to the Intramuscular Injection of Permethrin: An Uncommon Case Presentation

**DOI:** 10.7759/cureus.89524

**Published:** 2025-08-06

**Authors:** Juan M Gómez-Rodríguez, Jorge L Estévez-Arrizon, Luis E Ocampo-Guzmán, Oquitzin Flores-Palomares

**Affiliations:** 1 General Surgery, Hospital Regional Universitario de Colima, Colima, MEX; 2 Radiology, Hospital Regional Universitario de Colima, Colima, MEX

**Keywords:** abdominal wall, insecticides, intramuscular injection, necrotizing fasciitis, pyrethroids

## Abstract

Necrotizing fasciitis is a severe, rapidly progressing bacterial infection. We report a unique case in a 40-year-old woman with schizophrenia who developed necrotizing fasciitis after self-injecting permethrin intramuscularly into the lower abdomen. The patient presented with abdominal pain, fever, and a fluid collection on computed tomography. Surgical debridement and antibiotic therapy were performed, resulting in a favorable clinical evolution. The case suggests that permethrin induced local toxicity and tissue damage. Early diagnosis, prompt surgical intervention, and prevention of toxic substance misuse are essential.

## Introduction

Necrotizing fasciitis is a bacterial infection of the soft tissues that can cause local destruction of the epidermis, dermis, subcutaneous tissue, fascia, and muscle. It is characterized by rapid progression, potentially leading to sepsis, systemic toxicity, and multiorgan failure [1,2]. The term necrotizing fasciitis was first described by Joseph Jones, who based his findings on experiences during the American Civil War (1861-1865). He described it as an infection caused by "flesh-eating" bacteria and noted a mortality rate of approximately 50% [1,3].

It is considered a relatively rare condition, with a reported incidence ranging from 0.3 to 15 cases per 100,000 population [2,4]. The most significant risk factors include diabetes mellitus, advanced age, obesity, immunosuppression, intravenous drug use, and recent surgery [2,5]. As previously mentioned, the use of parenteral drugs is commonly associated with an increased risk of necrotizing fasciitis. However, no reports have been found documenting a relationship between this condition and exposure to pyrethroids.

Pyrethroids are a class of insecticides derived from the flowers of *Chrysanthemum cinerariifolium* and include commonly used pesticides such as deltamethrin, fenpropathrin, fenvalerate, bifenthrin, and permethrin. Pyrethroids are present in a wide variety of consumer products, including household insecticides, aerosols, pet shampoos, and mosquito repellents [6,7]. These insecticides primarily enter the body through skin contact, although they may also be absorbed via inhalation or ingestion. Permethrin, in particular, has been associated with a range of symptoms, including epidermal lesions, nausea, vomiting, abdominal pain, gastrointestinal mucosal irritation, and respiratory issues, among others [8]. The primary insecticidal mechanism of pyrethroids involves alteration of the kinetics of voltage-gated sodium channels. By prolonging the open state of these channels, pyrethroids increase sodium influx into neurons and slow the repolarization of the action potential [6]. Additionally, exposure to permethrin has been shown to activate caspases, potentially triggering apoptosis in human cells [9]. This mechanism may be associated with the tissue damage observed in the necrotizing fasciitis case presented in this report.

Necrotizing fasciitis is classified into three categories: Type I, representing polymicrobial infections; Type II, which involves Group A *Streptococcus*; and Type III, associated with marine Gram-negative bacteria [10]. In most cases, necrotizing fasciitis results from bacterial invasion through the epidermis, typically following trauma. This breach allows microorganisms to spread from the subcutaneous tissue to the deep fascial planes, a process facilitated by bacterial enzymes and toxins [11]. In anatomical regions such as the hands, feet, and scalp, fibrous septa within the subcutaneous tissue and fascia help contain infection. However, in the trunk and extremities, where such fibrous insertions are absent, the infection tends to spread more rapidly [11]. Classic clinical features of necrotizing fasciitis are often nonspecific and may include edema (75%), erythema (72%), severe pain (72%), tenderness (68%), fever (60%), and skin necrosis (38%). A hallmark sign is intense pain disproportionate to physical findings, which should raise clinical suspicion [8,9].

One of the most widely used diagnostic tools is the LRINEC (Laboratory Risk Indicator for Necrotizing Fasciitis) score. It incorporates six laboratory variables: C-reactive protein, white blood cell count, hemoglobin, serum sodium, serum creatinine, and glucose levels. A score of ≥8 is associated with a 75% probability of necrotizing fasciitis. Additional workup should include blood cultures for aerobic, anaerobic, and fungal organisms, Gram staining, and sensitivity testing to identify causative pathogens. Imaging studies such as computed tomography (CT) or ultrasound support diagnosis, typically revealing fluid collections and fascial thickening >4 mm [5]. Management follows standard principles for surgical infections, including source control, broad-spectrum antimicrobial therapy, fluid resuscitation, and vasopressors as needed based on the patient's hemodynamic status [5,11].

Surgical debridement is the cornerstone of treatment and must be performed early and aggressively. The fundamental principle is the complete excision of all necrotic tissue until healthy, bleeding tissue is encountered. Failure to achieve adequate debridement or delays in intervention are strongly associated with increased morbidity and mortality. Initiating surgical treatment within the first six hours may reduce mortality to 19%, while delays beyond this window may increase mortality to 32%. Re-exploration of affected tissue is recommended every 12-24 hours, depending on both local findings and systemic condition [5].

## Case presentation

A 40-year-old female patient with a two-year history of schizophrenia, untreated medically and without comorbidities, self-administered an intramuscular injection of insecticide (permethrin) in the lower hemiabdomen due to a delusional belief that she had a parasitic infestation. She presented to the emergency department with a 24-hour clinical course characterized by abdominal pain (Visual Analog Scale 7/10). She reported nausea without vomiting and an undocumented febrile peak. On physical examination, vital signs were as follows: blood pressure 110/80 mmHg, heart rate 95 bpm, temperature 38.5°C, and oxygen saturation 98%. She was alert with a Glasgow Coma Scale score of 15, oriented to person, place, and time, but showed indifference to surroundings. The patient was normocephalic, with pupils equal and reactive to light and no evidence of cervical lymphadenopathy on examination. Cardiopulmonary examination was unremarkable with well-ventilated lung fields. The abdomen was distended due to subcutaneous fat and tender to superficial and deep palpation in the right flank and hypogastric region, with a 3×3 cm area of induration and no signs of peritoneal irritation. Bowel sounds were normal. No crepitus was detected on palpation of the affected area.

Laboratory tests were requested as part of the initial diagnostic workup to identify parameters suggestive of systemic infection, organ dysfunction, or metabolic abnormalities. The results are presented in Table 1.

**Table 1:** Laboratory test results WBC: white blood cells; ALT: alanine aminotransferase; AST: aspartate aminotransferase; GGT: gamma-glutamyl transferase

Parameter	Result	Reference range	Units
Hemoglobin	12.5	12.0-15.0	g/dL
WBC	13.36	4.5-11.0	×10³/μL
Neutrophils	12.08	2.0-7.5	×10³/μL
Lymphocytes	0.86	1.0-4.0	×10³/μL
Platelets	425	150-450	×10³/μL
Glucose	93	80-100	mg/dL
Creatinine	0.7	0.7-1.2	mg/dL
Urea	23	17-49	mg/dL
ALT	21	9-52	U/L
AST	47	14-36	U/L
GGT	22	9-48	U/L
Total bilirubin	1.5	0.2-1.0	mg/dL
Direct bilirubin	0.3	0.1-0.3	mg/dL
Indirect bilirubin	0.3	<1.0	mg/dL

**Figure 1:**
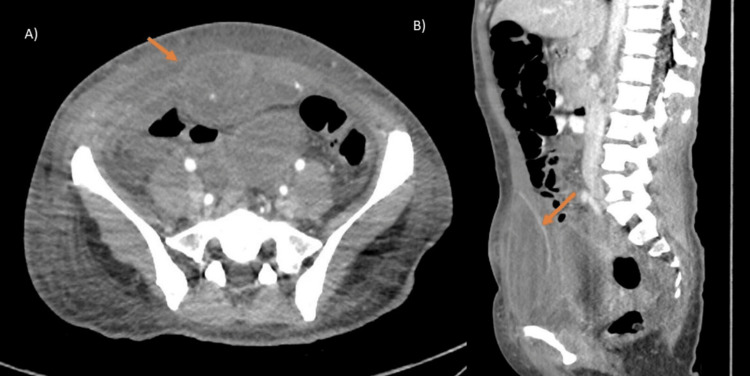
Arterial phase abdominopelvic CT scan: (A) axial view and (B) sagittal view A well-defined oval lesion (arrow) measuring 5×7 cm is observed in the hypogastric region at the right rectus abdominis, with fluid attenuation and peripheral enhancement after contrast administration. No additional abdominal lesions are identified. CT: computed tomography

Abdominopelvic CT showed, in the hypogastric region at the level of the right rectus abdominis muscles, a well-defined oval lesion measuring 5×7 cm with fluid attenuation. Post-contrast images demonstrated peripheral enhancement. No intra-abdominal lesions were identified (Figure 1A-1B).

CT played a key role in supporting the diagnosis of necrotizing fasciitis by revealing a fluid collection with peripheral enhancement consistent with severe soft tissue infection. These imaging findings, combined with the clinical presentation, guided the urgent decision for surgical exploration.

**Figure 2:**
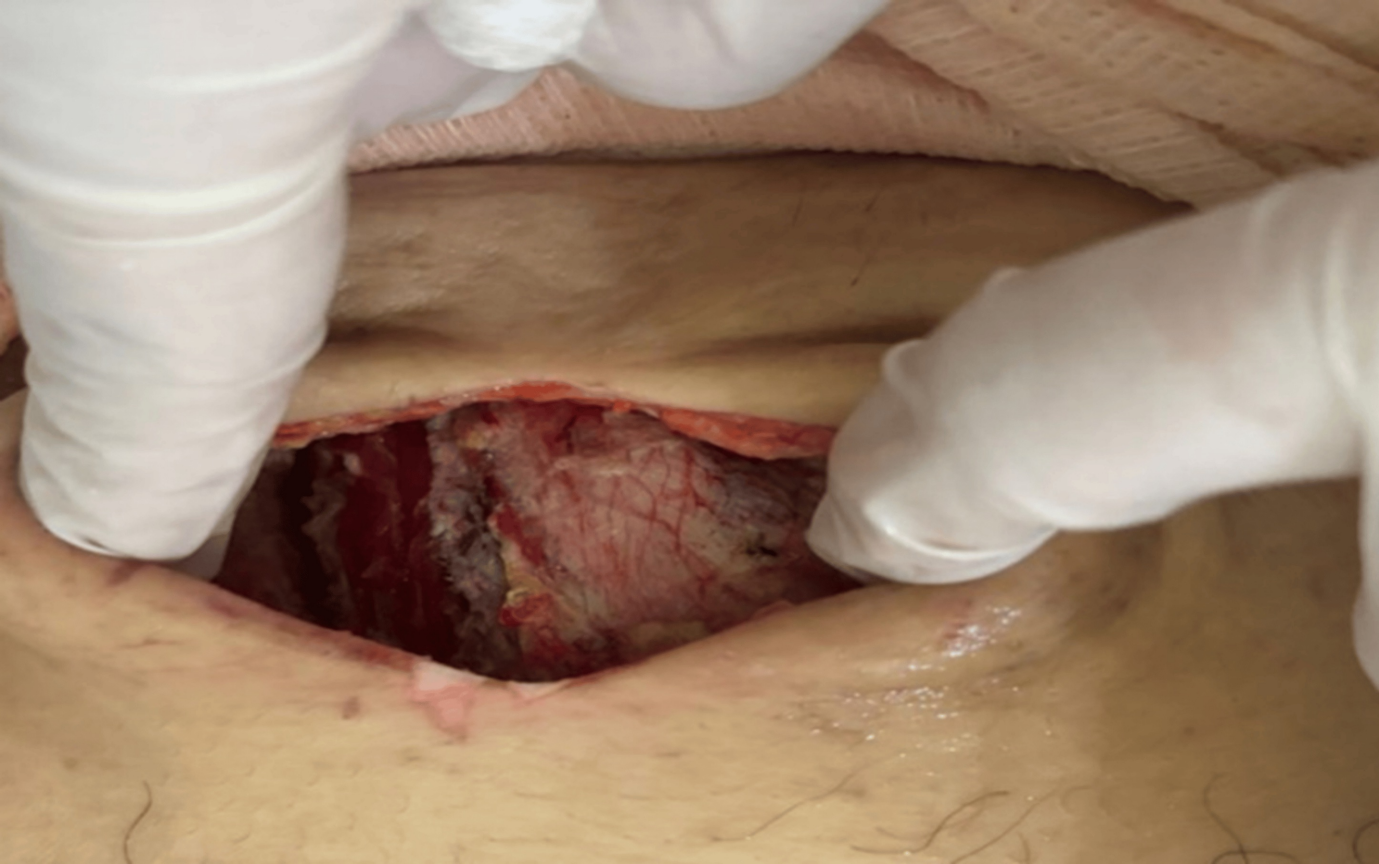
Postoperative wound in the right infraumbilical paramedian region showing active granulation tissue, with no evidence of necrosis and favorable healing progression

Based on these findings, the patient was taken to the operating room for debridement and drainage of the collection. A 12 cm paramedian incision was made in the infraumbilical region. Upon exploration, approximately 15 mL of purulent material and liquefied necrotic tissue were drained. Surgical findings revealed extensive involvement of the subcutaneous tissue, anterior aponeurosis, and rectus abdominis muscle, indicating a severe necrotizing soft tissue infection. Debridement and lavage with saline solution were performed, revealing lesion margins extending to the preperitoneal space without intraperitoneal involvement. The wound was left open for subsequent dressing changes. Initial antibiotic therapy consisted of ceftriaxone 1 g IV every 12 hours and metronidazole 500 mg IV every eight hours. A second surgical washout was performed 48 hours later, with ongoing bedside wound care. During a five-day hospital stay, the patient showed favorable progress with granulation tissue formation (Figure 2). The wound was closed by delayed primary intention using simple nylon 2-0 sutures. The patient was discharged with outpatient antibiotics, psychiatric follow-up, and wound care. At the 10-day follow-up after closure, the wound showed adequate healing and closure, and the patient was definitively discharged from surgical care to continue psychiatric management of her underlying condition.

## Discussion

Necrotizing fasciitis is an acute, severe, and rapidly progressing infection involving the skin, subcutaneous tissue, and sometimes muscle. Due to its rapid dissemination and potential lethality, timely diagnosis and treatment are crucial. Its onset may be favored by various factors, particularly those that alter tissue integrity [1,2]. In this context, intramuscular administration of permethrin represents an exceptionally rare complication, lending particular clinical relevance to this case. In this report, a patient developed necrotizing fasciitis following the intramuscular administration of permethrin, a commonly used pyrethroid insecticide. While dermal or inhalational exposure to this compound is well-documented [6-8], its direct introduction into muscle tissue is extraordinarily uncommon in the medical literature, underscoring the uniqueness of this clinical presentation.

The necrotizing fasciitis in this patient likely resulted primarily from the toxic effects of permethrin on the local tissues, which caused significant damage and inflammation. However, the possibility of bacterial contamination due to a non-sterile injection technique may have facilitated the infection, making the pathogenesis multifactorial.

Pyrethroids, such as permethrin, act on the nervous system by interfering with sodium channels in neuronal membranes [6]. However, their effects are not limited to the nervous system. Experimental studies have shown that they can also induce mitochondrial dysfunction, generate oxidative stress, and promote cellular apoptosis [9]. These mechanisms could have contributed to local tissue damage in this patient, creating an environment conducive to bacterial proliferation and necrotizing fasciitis development.

Additionally, permethrin has been reported to activate caspases, key enzymes in the apoptotic pathway [9]. This supports the hypothesis that the pro-apoptotic effects of pyrethroids may significantly contribute to cellular deterioration, facilitating the deep and extensive infection observed in this case.

This clinical case highlights the importance of recognizing the risks associated with the unconventional use of chemical substances like pyrethroids. Local or systemic toxicity resulting from such exposures can disrupt tissue homeostasis and promote the development of severe infections [2,5]. Furthermore, it underscores the need for a multidisciplinary approach in managing intoxicated patients and close monitoring for signs of complications [5,11]. Finally, documenting such events is crucial to expand knowledge about the adverse effects of these compounds and their potential association with severe infectious pathologies such as necrotizing fasciitis [1,2,5].

## Conclusions

This clinical case highlights the rare but severe complication of necrotizing fasciitis associated with intramuscular exposure to permethrin, a commonly used pyrethroid insecticide. The interaction of this compound with tissues can induce significant biological effects, such as mitochondrial dysfunction, oxidative stress, and cellular apoptosis, factors that likely facilitated bacterial proliferation. Although pyrethroids primarily affect the nervous system, this case demonstrates that their impact on the musculoskeletal system can also be detrimental. It is crucial to consider non-traditional exposures to pyrethroids as potential risks for tissue damage beyond their well-known neurotoxic effects. This case underscores the importance of a multidisciplinary approach to management, including early surgical intervention and appropriate antibiotic therapy, to ensure favorable clinical outcomes. Additionally, strict adherence to permethrin usage guidelines is essential, as intramuscular or parenteral administration is contraindicated due to the risk of tissue damage and systemic effects. Contact with mucous membranes or broken skin should be avoided, and caution is advised in children, pregnant women, and individuals with skin conditions. Finally, ongoing research into the toxic effects of pyrethroids is essential to better understand their risks and prevent associated complications.
